# Glue, Paste, and Papers

**Published:** 1872-01

**Authors:** 


### GLUE, PASTE, AND PAPERS.

You see an article in your paper; it pleases you very much; you feel as if you would like to read it again, and to preserve it. You lay the paper away, and at the end of a month, a year, or a dozen years, that paper remains on the shelf still, without ever having been referred to once. If you could have had a little paste at hand, have cut out the treasured article, and attached it to the blank leaf of any book much used, it might have been read and re-read with pleasure and profit many times.

You have many a time read a piece in the paper with great satisfaction; you purposed in your mind to cut it out, but business cares came in, and a month or a year later you have had occasion to refer to that article, and you find yourself deeply regretting that you could not lay your hand on it, and would very willingly pay the price of a year's subscription, to regain the lost piece. The date of a marriage, the obituary of a friend, complimentary notices of neighbors and fellow citizens, important events in town history — a multitude of "scraps" might be preserved with great satisfaction, not only for present reference, but for the years to come. Suppose a young person of twenty should get a scrap book, and make it a habit to cut out every piece in the paper which referred to schoolmates, friends, and fellow citizens, and keep it up for fifty years, with what deep emotions would the leaves be turned over after that long interval! and memories pleasurable and sad, how would they crowd the brain, and thoughts of cloud and sunshine come! The happy Christian death of some, how would they inspire the attempt to go and do likewise, to join them soon on the better shore! And if now and then the sad fate and failure of an old schoolmate is recorded, how would we drop a kind thought to his memory, and be thankful that we ourselves had not been drawn into the " same condemnation!"

The greatest impediment to all these is the want of a little paste; a cup of it, which can be made in a few minutes, will last for months, without moulding or smell or decomposition. Just here, our excellent neighbor and old time friend, the Editor of the

SCIENTIFIC AMERICAN, who gives us his admirable paper, so tidy looking, neat and clean, every week for a whole year for three dollars, every number abounding with solid information of all that is new and valuable in the shop, at the factory, on the farm, and about the house, comes to our relief and says, " Take one tablespoonful of flour, add gradually a pint of cold water, boil slowly, and stir well, to prevent burning, until it thickens. Keep boiling until it becomes thin, then add one teaspoonful of nitro-muriatic acid, and boil till it again thickens, when it is ready for use."

Speaking of sticky things, — you break a piece of china, or a vulgar plate; you don't like to throw it away, for only a small piece has been broken off, and yet you don't want to send half a mile or more to have it mended. Then make yourself a little

GLUE CEMENT

which will make even polished steel stick fast together, and keep it by you. " They say " that some old Turk found it out; but that neither adds to, nor diminishes its value.

Dissolve half a dozen bits of gum mastic, each as large as a pea, in as much alcohol as will make it a liquid.

In another vessel dissolve as much isinglass, previously softened with water, as will make a two ounce vial, that is, holding four tablespoonfuls, of strong glue, adding two small bits of gum ammoniac, which must be rubbed until it is dissolved in the isinglass mixture; then mix the whole with heat. When it is to be used again, set the vial in boiling water, until it is dissolved.

Then there is another kind of "stick;" the greatest trouble is in sticking too fast. It is your impecunious friend, whose feelings you do not wish to hurt; he is always borrowing a shilling, or even half a dollar, but always returns it. Just lend him five dollars, and he'll never stick you again. We know this is a good receipt, for we have tried it many a time and oft; it is a permanent cure; a second dose is never needed — cause why, he never comes near enough to you to repeat it.

But let us go back to the clipped newspapers. No man has an adequate idea of the real value of a well edited newspaper or magazine, until he is out at sea, or weather bound at a country inn, for a week or more at a time, compelling him to read every article, long as well as short. Take for example some of our New York exchanges, "Harper's Monthly Magazine," "The Galaxy," "Scribner's Monthly," or coming down to the weeklies, secular and religious, "The New York Evening Post," "The Independent," "The New York Evangelist," "The Baptist Examiner," "The Methodist," and "The Christian Advocate," and others; all of them abounding in every issue with the news from every part of the habitable globe; telling what is going on all over our own country, giving an idea of scientific, literary, and religious progress, with various items of information, practical, personal, and moral, which is really wonderful, and all for a few cents. Now the point is this: after you have read these, cut out what you want, paste it away; fold up the paper, tie a string around it, or wrap it in a piece of brown or white paper, paste on a two-cent stamp, and send it to some friend in the country; the further off, the more remote from railroad station or steamboat landing, the better, and the pleasure it gave you in the reading will be repeated many fold, in one, ten, or twenty other persons; for there are too many persons' families in the country who take no paper whatever; and among these a paper or periodical is highly prized, and is read, and loaned, and read over and over again for weeks and months together, or until a later arrival.

Surely this is a wiser method of disposing of our "papers," especially of our religious papers, than the "saving" them to look at again, until the worms and moths have eaten them up, or until they get so old and out of date as to make the tidy wife glad to get rid of them by using them for kindling. It even becomes a matter of conscience and of duty: ought a religious newspaper to be burned up with all its valuable moral and religious teachings? "Gather up the fragments, that nothing be lost," may have a wider meaning than the saving of little scraps of meat and bread. Two or three newspapers may be folded together and sent for two cents; but stick on the stamp with the paste above named, or a wafer, for it will not always adhere to a newspaper with its own gum. But in this connection it is not unworthy of mentioning how to make a

### SCRAP-BOOK.

Take any old account book, or old atlas, or other volume, count the number of leaves, divide by twenty; say there were a hundred leaves, this would give five leaves to each letter of the alphabet, for X, Y, and Z could be used as one, I and J for another, U, V, W, for another; then mark off the letters on the side of the page, or as in a dictionary: then paste every subject that begins with A on the A page, and so on. In this way, among other things, a great many valuable suggestions about health could be permanently preserved under their appropriate headings. Why, enough articles could be cut out of the newspapers which have been taken from HALL'S JOURNAL OF HEALTH, and from Dr. Hall's writings, to make a large and very valuable volume in a year, especially as everything taken from that source may be set down as practical, truthful, safe, and solid !

Under the letter M, put all the marriages of friends, adding at the time, in pencil, the year. Under the letter O, all the obituaries of dear kindred, and the friends of our youth. Under the letter B, all the facts and incidents which go to explain, exemplify, and substantiate Bible truths. Under the letter C, the charitable deeds and contributions of benevolent men; and under the letter L facts illustrating the dreadful horrors of the habit of liquor drinking, its certain end in moral, social, and pecuniary ruin; all to be read in after years by ourselves, and later on by our children, and in the long future by our grandchildren, as beacons and lighthouses for guidance, and warnings louder than a thousand thunders against the deep damnation of the habitual use of

THE INTOXICATING BOWL.

How in the world have we managed to connect paste, vulgar paste, with whiskey? but many a nice sort of a man, and woman too, would have skipped this article over, if we had headed it with "Liquor Drinking," or "Temperance." So we must essay to do good by stealth or unawares.

---

COLD KILLS. An incalculable amount of sickness and suffering, and multitudes of deaths, would be prevented every winter, if all were to make it imperative to keep comfortably warm all the time; then, taking cold is impossible.

